# Early Weight Loss After Sleeve Gastrectomy Selectively Improves Intermetatarsal Angle Without Affecting Hallux Valgus Angle

**DOI:** 10.3390/jcm15114086

**Published:** 2026-05-25

**Authors:** Furkan Türkoğlu, Soner Sarı, Elif Nur Gencer, Toygar Sağlam, Özgür Doğan

**Affiliations:** 1 Department of General Surgery, Aktif International Hospital, 41275 Kocaeli, Turkey; 2 Department of Orthopedics and Traumatology, Ankara Bilkent City Hospital, 06800 Ankara, Turkey; sarisnr97@gmail.com (S.S.); toygarsaglam96@gmail.com (T.S.); dr.ozgurdogan@gmail.com (Ö.D.); 3 Department of General Surgery, Tuzla State Hospital, 34947 Istanbul, Turkey; elifnurkocmar@yahoo.com

**Keywords:** bariatric surgery, hallux valgus, intermetatarsal angle, forefoot alignment, obesity, weight loss

## Abstract

**Background/Objectives**: While bariatric surgery is known to improve plantar pressure distribution and reduce foot-related symptoms, its impact on radiographic forefoot alignment parameters, such as the intermetatarsal angle (IMA) and hallux valgus angle (HVA), remains unclear, highlighting the need for further investigation. The aim of this study was to quantitatively evaluate changes of HVA and IMA in patients undergoing bariatric surgery and to determine whether weight loss-induced reductions in mechanical load are associated with differential changes of the forefoot. **Methods**: Weight-bearing anteroposterior foot radiographs of 102 patients who underwent laparoscopic sleeve gastrectomy were obtained preoperatively and at 6 months postoperatively. HVA and IMA were measured using standardized digital techniques. Changes in angular parameters were analyzed in relation to body mass index reduction. **Results**: Intra-observer reliability was excellent for all angular measurements (ICC: 0.987–0.999, *p* < 0.001). Radiographic evaluation demonstrated no significant change in HVA (*p* = 0.147), whereas IMA showed a significant decrease postoperatively (*p* < 0.001). No significant change was observed in HVA distribution (*p* = 0.341), and although the proportion of patients within the normal IMA range increased, this did not reach statistical significance (*p* = 0.091). Correlation analysis revealed significant associations of postoperative angles with age and body weight parameters (*p* < 0.05), while no significant relationship was found between changes in body weight or BMI and angular measurements (*p* > 0.05). **Conclusions**: Sleeve gastrectomy significantly improves biomechanically driven aspects of forefoot alignment (decrease in IMA), while structural deformities (HVA) remain unchanged in the short-term. These findings highlight that obesity-related forefoot pathology is predominantly linked to altered load distribution rather than fixed structural deformity, suggesting that radiographic parameters reflecting biomechanical loading are more sensitive to weight loss.

## 1. Introduction

Obesity is an increasingly prevalent chronic disease with complex multisystemic effects [1]. Beyond its well-known metabolic and cardiovascular consequences, obesity exerts significant negative impacts on the musculoskeletal system, often leading to pain, functional limitation, and reduced mobility, thereby restricting patients’ ability to perform daily activities [2,3,4]. As is comprehensively evidenced by the extant literature, obesity is associated with several musculoskeletal sequelae [3,5,6]. Of these, foot pathologies are of particular significance, given their direct impact on weight-bearing, gait mechanics, and overall functional capacity [7,8]. Forefoot pathologies such as hallux valgus are particularly prevalent in obese individuals, who are also predisposed to a wide range of foot-related disorders, including but not limited to increased plantar sensitivity, decreased medial longitudinal arch height, decreased sagittal balance, excessive pronation, hindfoot valgus alignment, and overuse conditions such as tendinitis and plantar fasciitis [9,10,11,12]. Hallux valgus (HV) is defined as a progressive three-dimensional forefoot deformity of the first metatarsophalangeal joint [13,14,15,16,17], with a reported prevalence ranging from 11–22.7% [17]. Beyond its epidemiological burden, HV carries substantial biomechanical consequences: deformity of the first metatarsophalangeal joint alters load transmission along the medial column, impairs push-off mechanics during the terminal stance phase of gait, and promotes compensatory loading of the lesser metatarsals [18,19,20].

The treatment for hallux valgus is determined by several factors, including the severity of deformity, the intensity of symptoms, functional impairment, and the patient’s expectations. First-line treatment typically involves the elimination of existing etiological factors, the selection of appropriate footwear, and the use of temporary orthotic devices. The objective of conservative measures is twofold: firstly, to redistribute forefoot load, and secondly, to alleviate pain, without correcting the underlying bone deformity. In cases of moderate-to-severe deformities, the surgical intervention may involve distal metatarsal, proximal metatarsal, combined osteotomies, or fusion (Lapidus) procedures, depending on the extent of the deformity. The objective of surgical intervention is the restoration of HVA and IMA [13,14,15,16,17].

Forefoot structures play a key role in load transmission during weight-bearing and are exposed to increased mechanical stress in obese individuals [10,21]. Excess body weight alters load distribution across the forefoot, with a substantial portion transmitted through the first ray and hallux, potentially leading to malalignment. Although both parameters are used in combination to diagnose hallux valgus deformity, the intermetatarsal angle (IMA), which represents the divergence between the first and second metatarsals, is more closely associated with dynamic forefoot load distribution, whereas the hallux valgus angle (HVA), which quantifies the angular deviation of the hallux at the metatarsophalangeal joint, is generally regarded as a marker of a more fixed structural deformity [22].

Bariatric surgery has emerged in recent years as a safe and effective treatment modality, yielding consistent and sustained weight loss outcomes [23]. Beyond weight reduction, bariatric procedures contribute to the improvement or partial reversal of several obesity-related adverse effects, including metabolic dysfunction, systemic inflammation, and obesity-associated comorbidities [4,24]. A growing body of evidence indicates that bariatric surgery leads to improvements in foot-related symptoms and patient-reported outcomes in obese individuals [24]. Reductions in both the frequency and severity of pain have been observed, accompanied by improved functional capacity and activities of daily living [25,26]. Despite these symptomatic improvements, the extent to which radiographic forefoot parameters are modified following bariatric surgery, particularly during the acute weight-loss period, remains largely unknown.

It remains unclear whether HVA and IMA are modifiable through early weight loss or whether clinical improvements are accompanied by measurable radiographic changes. Accordingly, the aim of this study was to quantitatively evaluate changes in key forefoot radiographic parameters, the HVA and IMA, in patients undergoing bariatric surgery, and to determine whether early weight loss-induced reductions in mechanical load are associated with differential changes in these parameters. To date, no study has evaluated differential changes in HVA and IMA following bariatric surgery-induced weight loss in the short-term, using standardized weight-bearing radiographs. The hypothesis is that IMA, as a load-sensitive parameter, would demonstrate greater and earlier improvement following sleeve gastrectomy in comparison to HVA, which reflects more structurally fixed deformity.

## 2. Materials and Methods

### 2.1. Study Design and Patient Selection

This retrospective study included patients who underwent primary laparoscopic sleeve gastrectomy for the treatment of obesity at a single tertiary care center. To minimize inter-operator variability, all procedures were performed by the same experienced bariatric surgeon (FT). Ethical approval was obtained from the institutional review board prior to study initiation (TABED 1-26-2469). All patients who underwent primary laparoscopic sleeve gastrectomy performed by the same surgeon in the predefined study period were eligible for inclusion. Patients who underwent other bariatric procedures or revision bariatric surgery were excluded. Additional exclusion criteria comprised a history of prior foot or ankle surgery, previous trauma, rheumatologic disease, neuromuscular disorders affecting gait, congenital, or severe structural foot deformities, and incomplete clinical or radiological data. Finally, all anteroposterior foot radiographs that were reviewed in this study were obtained with strict adherence to standardized protocols that have been outlined in the literature for weight-bearing dorsoplantar foot imaging. This imaging modality is regarded as indispensable for the precise evaluation of foot alignment, as non-weight-bearing projections are deemed inadequate due to the absence of functional positioning of the osseous structures. The quality of the images was evaluated according to well-established radiographic criteria, encompassing the adequate visualization of metatarsal alignment, intermetatarsal spacing, and the opening of the intertarsal space between the medial and intermediate cuneiforms. Patients whose radiographs did not meet these predefined imaging quality requirements were excluded from the study.

Following the application of the inclusion and exclusion criteria, data from 102 prospectively followed patients were retrospectively analyzed. All procedures involving human participants were conducted in accordance with the ethical standards of the institutional research committee and in line with the Declaration of Helsinki and its subsequent revisions. Generative artificial intelligence (DeepL Translator, Free Version, DeepL SE, Cologne, Germany). was used solely for basic language editing purposes, including spelling and punctuation corrections. No large language models or other generative AI tools were used for the generation of text, data, or visual materials, nor did they contribute to the study design, data collection, analysis, or interpretation. This observational retrospective study was designed in accordance with the STROBE guidelines.

### 2.2. Surgical Technique and Routine Follow-Up

Primary laparoscopic sleeve gastrectomy was performed in all patients using standardized surgical techniques and perioperative management protocols as described in the literature, including but not limited to anesthesia, antibiotic prophylaxis, and thromboprophylaxis [27,28]. During the postoperative period, all patients were monitored according to standardized protocols, including drain output assessment and wound care. Furthermore, all patients were followed by the same multidisciplinary team, including a dietitian and a physiotherapist, and were managed according to a standardized postoperative nutritional and rehabilitation program.

### 2.3. Clinical and Radiographical Data

Demographic and clinical data, including age, sex, and patient height (cm), were collected from medical records. Body weight (kg) and body mass index (BMI, kg/m^2^) were recorded preoperatively and at postoperative follow-up, and changes in these parameters were calculated as the difference between preoperative and postoperative values. Follow-up duration was recorded in weeks for all patients.

Radiographic evaluation was performed using weight-bearing anteroposterior foot radiographs to ensure physiological loading conditions. Forefoot alignment parameters included the hallux valgus angle, defined as the angle between the longitudinal axis of the first metatarsal and the longitudinal axis of the proximal phalanx of the hallux, and the intermetatarsal angle, defined as the angle between the longitudinal axes of the first and second metatarsals. The longitudinal axis of each metatarsal was determined by bisecting the diaphysis at two points and connecting these midpoints [22]. In addition to continuous measurements, HVA and IMA were categorized based on commonly used clinical thresholds as normal (<15°) or increased (≥15°) for HVA and normal (<9°) or increased (≥9°) for IMA. All measurements were performed using standardized digital imaging techniques by a single observer, and intra-observer reliability was assessed using intraclass correlation coefficients (ICCs) based on repeated measurements with three month intervals (Table 1). The interobserver reliability of the measurements was not assessed, as both HVA and IMA are well-validated radiographic measurements with established high interobserver agreement in the literature [13]. The reliability of the single observer responsible for all radiographic assessments in this study was measured to ascertain the consistency of that observer’s work.

### 2.4. Statistical Analysis

Statistical analyses were conducted to compare preoperative and postoperative values and to evaluate relationships between variables. Continuous variables were expressed as mean ± standard deviation or median (minimum–maximum) depending on distribution, and normality was assessed separately for each variable using the Shapiro–Wilk test. The paired samples t-test or Wilcoxon signed-rank test were used for preoperative and postoperative comparisons as appropriate, while categorical variables were compared using the chi-square test or Fisher’s exact test. Correlation analyses were performed using Spearman’s rank correlation coefficient to evaluate the relationship between radiographic parameters and demographic and anthropometric variables. All statistical analyses were conducted using SPSS version 26.0 (IBM Corp., Armonk, NY, USA), and a *p*-value of <0.05 was considered statistically significant. All reported confidence intervals were calculated as 95% confidence intervals. The intraclass correlation coefficient (ICC) was utilized to evaluate the intra-observer reliability of radiographic angular measurements. The interpretation of ICC values was as follows: values below 0.50 indicated poor reliability, 0.50–0.75 moderate reliability, 0.75–0.90 good reliability, and values above 0.90 excellent reliability. Given the retrospective nature of the study, a priori sample size calculation was not performed. The sample size was determined by the number of patients fulfilling the eligibility criteria within the study period. A post hoc power analysis was conducted to assess the statistical adequacy of the sample, confirming adequate power (>80%) for the primary outcomes assessed.

## 3. Results

A total of 102 patients were included in the study, with a median age of 41 years, a predominance of female patients (79.4%), and a median follow-up duration of 26 weeks (Table 2). Intra-observer reliability analysis demonstrated excellent agreement for all angular measurements, with ICC ranging from 0.987 to 0.999 (*p* < 0.001 for all parameters) (Table 1). Following bariatric surgery, patients exhibited a significant reduction in body weight and body mass index, with median body weight decreasing from 113.05 kg to 87.8 kg and BMI from 42 kg/m^2^ to 32.5 kg/m^2^ (*p* < 0.001 for both) (Table 2).

**Figure 1:**
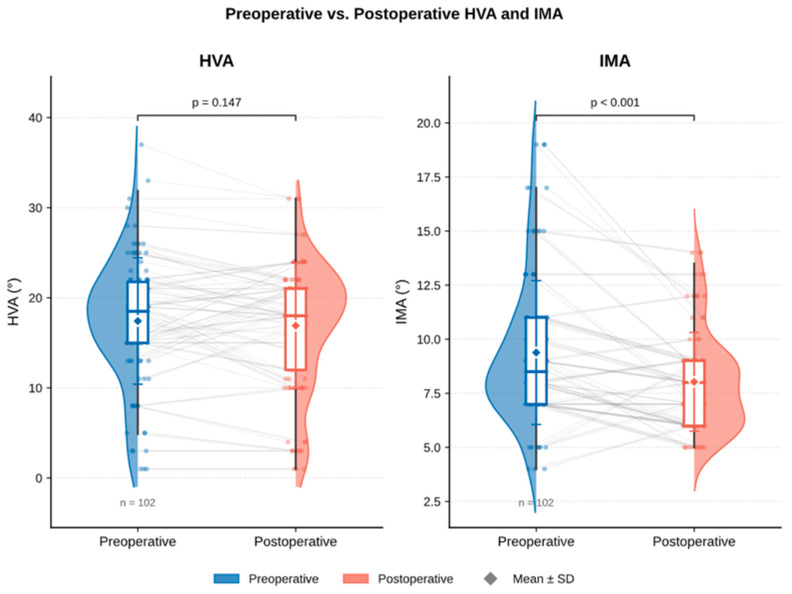
Figures demonstrate the preoperative and postoperative changes in HVA and IMA. Paired raincloud plot showing the change of HVA postoperatively (*p* = 0.147) on the left, and Paired raincloud plot showing the change of IMA postoperatively (*p* < 0.001) on the right.

In terms of radiographic parameters, no statistically significant change was observed in the HVA (*p* = 0.147), whereas the IMA demonstrated a statistically significant decrease following surgery (*p* < 0.001) (Figure 1) (Table 2). When categorized according to normal reference ranges, no significant change was observed in HVA distribution (*p* = 0.341), and although the proportion of patients within the normal range for IMA increased postoperatively, this change did not reach statistical significance (*p* = 0.091) (Table 3).

Correlation analysis revealed that both postoperative HVA and IMA were negatively correlated with age (*p* = 0.005 and *p* = 0.001, respectively), preoperative body weight (*p* = 0.003 and *p* = 0.004), preoperative BMI (*p* = 0.017 and *p* = 0.015), and postoperative body weight (*p* < 0.001 for both) (Table 4). Strong positive correlations were observed between preoperative and postoperative angular measurements, with postoperative HVA strongly correlating with preoperative HVA (r = 0.690, *p* < 0.001), while postoperative IMA showed a strong correlation with preoperative IMA (r = 0.642, *p* < 0.001) and moderate correlation with preoperative HVA (r = 0.434, *p* < 0.001) (Table 4). No significant correlations were identified between changes in body weight or BMI and postoperative forefoot angles (*p* > 0.05 for all) (Table 4).

## 4. Discussion

In obese individuals, foot-related pathologies exert significant physical, psychological, and social burdens, and their improvement following bariatric surgery represents an important positive outcome that contributes to enhanced quality of life [5,26,29]. These disorders occupy a particularly important position due to their direct involvement in weight-bearing, gait mechanics, and functional performance [8,30,31]. Given that IMA and HVA reflect both mechanical loading conditions and structural characteristics, they provide a useful framework for investigating the effects of early weight loss after sleeve gastrectomy on forefoot alignment. Previous studies have demonstrated that hallux valgus-related radiographic and clinical parameters are closely associated with altered forefoot biomechanics, including abnormal gait patterns and plantar pressure distribution during walking [32,33]. However, radiographic changes in these parameters following bariatric surgery have rarely been investigated. The most notable finding of this study was that although no statistically significant change was observed in HVA following surgery (*p* = 0.147), IMA demonstrated a significant decrease (*p* < 0.001), while neither parameter showed a significant shift in distribution relative to normal reference ranges (HVA, *p* = 0.341; IMA, *p* = 0.091).

The most striking finding of this study is the differential response of forefoot parameters to early weight loss. Our findings suggest that early weight loss may differentially affect forefoot alignment parameters. While the decrease in IMA may be attributed to reduced plantar loading and improved biomechanical distribution, the lack of change in HVA likely reflects its more structural and less reversible nature. On the other hand, overinterpretation should be avoided in terms of IMA, as no biomechanical assessment or gait analysis was performed in the present study. Although a statistically significant reduction in IMA was observed following rapid weight loss after sleeve gastrectomy, the absolute magnitude of change was modest. Consequently, the clinical significance of this finding should be interpreted with caution. It is acknowledged that minor angular disparities in radiographic measurements may be subject to partial overlap with inherent measurement variability. This is particularly evident in retrospective assessments influenced by patient positioning, radiographic technique, and observer-dependent factors. Consequently, while the observed change may suggest that early weight loss-associated reductions in mechanical loading can influence forefoot alignment, this finding should not be interpreted as evidence of a clinically meaningful structural correction at this stage. Instead, it may be indicative of a subtle mechanobiological response that warrants further investigation in prospective studies employing standardized imaging protocols, and correlation with clinical and functional outcomes.

The lack of change in HVA may further be explained by its multifactorial etiology, including ligamentous laxity, joint degeneration, and long-standing structural adaptation, which are unlikely to reverse in the short-term despite reduced mechanical load [34]. Furthermore, HVA is also influenced by sesamoid position and the tension of the adductor hallucis tendon. Once sesamoid subluxation becomes established, it represents a relatively rigid structural deformity that is unlikely to be altered by a simple reduction in mechanical loading alone. Our findings are consistent with previous literature suggesting that hallux valgus is not strongly associated with obesity-related mechanical loading [5], which may explain the absence of a significant change in HVA in our study. Similarly, Menz et al. [34] found no significant association between obesity and either the development or progression of hallux valgus. In addition, the relatively short follow-up period in the present study may have limited the ability to detect potential changes in HVA, as structural parameters may require longer periods to demonstrate measurable adaptation.

In addition to the differential changes observed in forefoot alignment parameters, the correlation analysis provides further insight into the factors that may influence postoperative alignment. Both postoperative HVA and IMA demonstrated significant associations with age and body weight-related parameters, suggesting that patient-specific characteristics may contribute to the final alignment. Notably, strong correlations were observed between preoperative and postoperative angular measurements, which may indicate that baseline deformity plays an important role in determining postoperative outcomes. These findings may suggest that beyond the effects of weight loss, the initial severity of deformity could remain an important determinant of final radiographic alignment. Therefore, postoperative forefoot alignment appears to be influenced by both biomechanical unloading and pre-existing anatomical factors. Whilst the hypothesis that structural deformities may not improve in the early period following weight loss, whereas parameters related to dynamic forefoot load distribution may show improvement, appears to be a plausible and testable theory, the absence of a significant correlation between the degree of weight/BMI reduction and postoperative IMA suggests that a direct relationship cannot be established. The forefoot alignment is likely determined by a complex interplay of multiple biomechanical and anatomical factors that extend beyond body weight alone. Consequently, the proposed mechanical interpretation should be regarded as exploratory rather than confirmatory, and prospective clinical studies with larger cohorts and longer follow-up periods are required to more clearly elucidate the causal relationship between weight loss and structural changes in forefoot alignment.

Previous research has indicated that despite improvements in plantar pressure distribution and quality of life following bariatric surgery, foot-related pathologies may persist or even emerge after weight loss [24]. This suggests that not all foot-related alterations are fully reversible and supports the concept that structural components may be less responsive to weight reduction. In line with this, our findings demonstrate that while mechanically influenced parameters showed improvement, structural alignment remained largely unchanged. Hallux valgus is inherently a three-dimensional deformity characterized not only by transverse plane deviation, but also by rotational and multiplanar structural alterations that may not be fully captured by conventional two-dimensional radiographic measurements [13,35]. The weight reduction consequent to sleeve gastrectomy has the potential to decrease axial loading across the forefoot, modify plantar pressure distribution, and to a certain extent restore sagittal foot balance [36]. This, in turn, has the capacity to influence dynamic force transmission through the first ray. Within this biomechanical context, the observed reduction in IMA may suggest that metatarsal alignment is relatively sensitive to changes in mechanical loading. Conversely, HVA may be indicative of a more established structural deformity influenced by chronic soft-tissue imbalance, capsuloligamentous adaptation, and rotational malalignment, which may be less responsive to short-term weight reduction. On the other hand, it is imperative to acknowledge that the present study concentrated exclusively on radiographic changes, and that the clinical implications of these findings should be interpreted separately. Furthermore, given that the study population consisted exclusively of obese individuals, whose baseline foot morphology and biomechanical characteristics are not expected to mirror those of the general population, direct interpretation should be made with caution. It should be noted that obese foot, which is chronically collapsed, has been associated with increased plantar loading in the midfoot and forefoot regions, a flatter foot posture, and high plantar pressure. As indicated by the extant literature, rapid weight loss and associated offloading have been documented to result in improvement in the plantar arch and significant improvement in the sagittal balance [10,36,37,38]. Further prospective studies incorporating functional assessments, plantar pressure analysis, and dynamic biomechanical measurements are necessary to provide a more objective clarification of the relationship between first-ray load transfer, forefoot balance, and changes in IMA and HVA following substantial weight loss.

The findings of this study may have several practical implications for clinical practice. Firstly, the differential responses of IMA and HVA to weight loss are directly relevant to clinical decision-making processes, in the context of surgical planning. Specifically, the substantial decrease in IMA observed following weight reduction indicates that while the deformity may not be fully resolved, a degree of correction or alleviation may be achieved. This finding may have practical implications for orthopedic surgeons, as preoperative weight loss in obese patients with hallux valgus could potentially influence the choice and extent of corrective osteotomy required. Secondly, the absence of a substantial change in HVA suggests that the structural deformity at the metatarsophalangeal joint is unlikely to improve with weight loss alone, at least in the early postoperative period. This observation is clinically significant for the purpose of providing appropriate patient counseling, particularly for individuals undergoing bariatric surgery with expectations of complete resolution of musculoskeletal complaints. Finally, the absence of a substantial correlation between the extent of weight reduction and postoperative angular values indicates that absolute postoperative body weight, rather than the magnitude of weight reduction alone, may serve as a more significant predictor of forefoot biomechanics. This observation underscores the potential significance of achieving and sustaining target weight thresholds, as opposed to concentrating exclusively on the extent of weight reduction.

This study has several limitations that should be acknowledged. First, its retrospective design inherently introduces the potential for selection bias. Second, the relatively short follow-up period may have been insufficient to detect longer-term structural adaptations, particularly in parameters such as the HVA, which may require a more prolonged period to demonstrate measurable change. Third, the single-center design and relatively limited sample size may restrict the generalizability of the findings. Fourth, potentially important confounding factors, including footwear habits, physical activity level, and individual biomechanical variations, were not systematically assessed. Another important limitation is the inability to perform robust multivariable regression analyses due to the limited sample size and incomplete availability of relevant confounding variables, thereby precluding adequate adjustment for residual confounding. Fifth, clinical and functional outcomes were not evaluated, limiting the ability to determine whether the observed radiographic changes translated into meaningful patient-reported or functional improvements. In this matter, a further notable limitation is the absence of a comprehensive preoperative foot examination, particularly the lack of assessment of first ray hypermobility. First ray hypermobility, defined as excessive sagittal plane motion involving the first metatarsal, the first tarsometatarsal joint, and the medial cuneiform, is a well-recognized biomechanical factor in the pathogenesis of hallux valgus and has been associated with increased IMA [13,14,15,16,17]. The omission of this parameter limits the ability to determine whether the observed postoperative changes in IMA following rapid weight loss were influenced by pre-existing variations in first ray stability. Prospective studies evaluating the relationship between rapid weight loss and forefoot radiographic alignment should incorporate standardized preoperative assessment of first ray hypermobility to provide a more comprehensive understanding of forefoot biomechanics in obese patients. It should also be noted that the absence of a non-surgical control group limits the ability to distinguish the independent effects of weight loss from other postoperative factors, such as behavioral modifications, changes in physical activity, or rehabilitation-related adaptations. Finally, the reliance on two-dimensional radiographic measurements may not fully capture the complex three-dimensional biomechanics of the forefoot, while the absence of plantar pressure analysis or gait assessment precludes a more comprehensive evaluation of functional biomechanical changes. Despite these limitations, the study provides preliminary insights into the early radiographic effects of rapid postoperative weight loss on forefoot alignment and may serve as a foundation for future prospective investigations.

## 5. Conclusions

Rapid weight loss following sleeve gastrectomy was associated with modest early changes in certain forefoot radiographic alignment parameters, particularly the intermetatarsal angle, while the structurally based hallux valgus angle remained unchanged in the short-term. The differential behavior of these radiographic parameters following rapid postoperative weight loss may suggest that certain alignment characteristics are more responsive to early biomechanical adaptation than others; however, such changes are likely influenced by multiple interacting factors, including baseline deformity severity, load distribution patterns, and the intrinsic biomechanical properties of the foot. Components related to structural deformity may require longer follow-up or additional interventions to demonstrate clinically meaningful change. These findings provide preliminary insight into the differential effects of weight loss on forefoot alignment and may contribute to future clinical evaluation and management strategies.

## Figures and Tables

**Table 1:** Intra-observer reliability of the angular parameters.

	Intra-Observer Reliability	
	ICC	*p*
Preoperative Hallux Valgus Angle	0.998	<0.001
Preoperative Intermetatarsal Angle	0.990	<0.001
Postoperative Hallux Valgus Angle	0.999	<0.001
Postoperative Intermetatarsal Angle	0.987	<0.001

ICC: Interclass correlation; *p*: Statistical significance value.

**Table 2:** Demographic profile of the patients and anthropometric and forefoot angular parameters before and after laparoscopic sleeve gastrectomy.

		Median (IQR)	Range (Min–Max)	*p*
Age (years)		41 (17)	18–63	N/A
Gender	Female	81 (79.4%)		
	Male	21 (20.6%)		
Length (cm)		160.5 (9)	146–180	
Follow-up (weeks)		26 (1)	23–28	
Body Weight Change (kg)		23.85 (7.5)	12.5–37.7	
Body Mass Index Change (kg)		9.7 (2.7)	4.1–12.8	
Body Weight (kg)	Preop	113.05 (28.1)	77–152	<0.001
	Postop	87.8 (21)	57–119	
Body Mass Index (kg/m^2^)	Preop	42 (8.5)	35.1–51.1	<0.001
	Postop	32.5 (5.7)	25.3–41	
Hallux Valgus Angle (°)	Preop	18.5 (7)	1–37	0.147
	Postop	18 (9)	1–31	
Intermetatarsal Angle (°)	Preop	8.5 (4)	4–19	<0.001
	Postop	8 (3)	5–14	

IQR: Interquartile range, Min: Minimum, Max: Maximum, *p*: statistical significance value; N/A: Not applicable, Preop: Preoperative, Postop: Postoperative.

**Table 3:** Changes in the distribution of forefoot angular parameters before and after laparoscopic sleeve gastrectomy.

		Preoperative	Postoperative	*p*
Hallux Valgus Angle (NR: <15°)	Within Range	24 (23.5%)	30 (29.4%)	0.341
	Out of Range	78 (76.5%)	72 (70.6%)	
Intermetatarsal Angle (NR: <9°)	Within Range	51 (50%)	63 (61.8%)	0.091
	Out of Range	51 (50%)	39 (38.2%)	

*p*: Statistical significance value; NR: Normal range.

**Table 4:** Correlation between preoperative and postoperative forefoot angular parameters and demographic and anthropometric characteristics.

	Postoperative Hallux Valgus Angle	Postoperative Intermetatarsal Angle
Age	*p* = 0.005 (r = −0.279)	*p* = 0.001 (r = −0.333)
Patient Height	*p* = 0.169	*p* = 0.160
Preoperative Body Weight	*p* = 0.003 (r = −0.296)	*p* = 0.004 (r = −0.280)
Preoperative Body Mass Index	*p* = 0.017 (r = −0.235)	*p* = 0.015 (r = −0.240)
Postoperative Patient Weight	*p* < 0.001 (r = −0.353)	*p* < 0.001 (r = −0.361)
Postoperative Body Mass Index	*p* = 0.089	*p* = 0.025 (r = −0.222)
Weight Change	*p* = 0.326	*p* = 0.066
Body Mass Index Change	*p* = 0.310	*p* = 0.288
Preoperative Hallux Valgus Angle	*p* < 0.001 (r = 0.690)	*p* < 0.001 (r = 0.434)
Preoperative Intermetatarsal Angle	*p* = 0.021 (r = 0.229)	*p* < 0.001 (r = 0.642)
Postoperative Hallux Valgus Angle	N/A	*p* = 0.002 (r = 0.306)
Postoperative Intermetatarsal Angle	*p* = 0.002 (r = 0.306)	N/A

*p*: Statistical significance value; r: Spearman correlation coefficient; N/A: Not applicable. The values in the cells represent *p*-values indicating statistical significance. The Spearman correlation coefficient is shown for cells where the *p*-value is less than 0.05.

## Data Availability

The datasets generated and/or analyzed during the current study are stored in a private repository and are not publicly available; however, they may be obtained from the corresponding author upon reasonable request.

## Abbreviations

The following abbreviations are used in this manuscript:

HVA	Hallux valgus angle
IMA	Intermetatarsal angle
BMI	Body mass index
ICC	Interclass correlation
NR	Normal range
N/A	Not applicable
